# Enrichment of plasma extracellular vesicles for reliable quantification of their size and concentration for biomarker discovery

**DOI:** 10.1038/s41598-020-78422-y

**Published:** 2020-12-07

**Authors:** Marija Holcar, Jana Ferdin, Simona Sitar, Magda Tušek-Žnidarič, Vita Dolžan, Ana Plemenitaš, Ema Žagar, Metka Lenassi

**Affiliations:** 1 Institute of Biochemistry and Molecular Genetics, Faculty of Medicine, University of Ljubljana, Ljubljana, Slovenia; 2 Department of Polymer Chemistry and Technology, National Institute of Chemistry, Ljubljana, Slovenia; 3 Department of Biotechnology and System Biology, National Institute of Biology, Ljubljana, Slovenia

**Keywords:** Biomarkers, Biomarkers, Cell biology

## Abstract

Human plasma is a complex fluid, increasingly used for extracellular vesicle (EV) biomarker studies. Our aim was to find a simple EV-enrichment method for reliable quantification of EVs in plasma to be used as biomarker of disease. Plasma of ten healthy subjects was processed using sedimentation rate- (sucrose cushion ultracentrifugation—sUC) and size- (size exclusion chromatography—SEC) based methods. According to nanoparticle tracking analysis (NTA), asymmetrical flow field-flow fractionation coupled to detectors (AF4-UV-MALS), miRNA quantification, transmission electron microscopy and enzyme-linked immunosorbent assay, enrichment of EVs from plasma with sUC method lead to high purity of EVs in the samples. High nanoparticle concentrations after SEC resulted from substantial contamination with lipoproteins and other aggregates of EV-like sizes that importantly affect downstream EV quantification. Additionally, sUC EV-enrichment method linked to quantification with NTA or AF4-UV-MALS is repeatable, as the relative standard deviation of EV size measured in independently processed samples from the same plasma source was 5.4% and 2.1% when analyzed by NTA or AF4-UV-MALS, respectively. In conclusion, the sUC EV-enrichment method is compatible with reliable measurement of concentration and size of EVs from plasma and should in the future be tested on larger cohorts in relation to different diseases. This is one of the first studies using AF4-UV-MALS to quantify EVs in blood plasma, which opens new possible clinical utility for the technique.

## Introduction

Extracellular vesicles (EVs) are phospholipid bilayer-enclosed nanosized particles (50–1000 nm) secreted by all cell types^1^. EVs’ molecular cargo, size and concentration reflect the state of their cell-of-origin^2^, making them ideal candidates for biomarkers of various pathological conditions^3–6^. EVs are found in all body fluids, although blood is the most frequently used for biomarker research^7^. Peripheral venous blood requires a minimally invasive collection procedure and its dynamic composition closely relates to the (pato)physiologic changes in the organism^8^. Blood is a highly complex fluid involved in maintaining homeostasis in the body. Plasma, the liquid part of the blood, contains a plethora of extracellular nanoparticles, among them EVs, which can have common characteristics^9^. By far the most abundant nanoparticles are different proteins and protein complexes, but even circulating lipoproteins outnumber blood-EVs for six to seven orders of magnitude^7,10^. If present, enveloped viruses, such as retroviruses, are also budding from the cells and contribute to blood nanoparticles^1^. Despite increasing scientific and clinical interest^7^, lack of a standardized method of EVs enrichment from plasma hinders reliable and comparable quantification of blood-derived EVs.

Two of the most often used methods for EVs enrichment are: (1) ultracentrifugation (UC), which separates particles based on their sedimentation rate (dependent on particle size, shape and flotation density), and (2) size exclusion chromatography (SEC), which separates particles based on their size^11,12^. UC usually follows a few initial steps of differential centrifugation at lower speeds, which ensure gradual removal of cells, cellular debris, and larger soluble protein/lipoprotein aggregates, whereas UC with acceleration at ≥ 100,000×*g*, helps to enrich EVs and any other nanoparticles in the sample. With an addition of density gradient or cushion of denser solution (like sucrose) to the UC step, efficacy of enrichment process and purity of EVs can be increased and potential mechanical damage to the vesicles reduced^13^. SEC is another simple and increasingly popular column-based method for EV enrichment^14^. Successful separation should lead to EV-enriched fractions, free of non-EV contaminants, with EVs retaining their structure and physiological function^15^, but SEC is unable to separate EVs from other nanoparticles of similar size^16,17^. Combining different techniques would help, but high numbers and relatively low starting volumes of samples, typically analyzed in clinical environments, do not support the use of expensive and lengthy multistep EV-enrichment processes. Therefore, we need a simple method for enrichment of EVs from small blood volumes, which can easily be implemented for consistent and high throughput use.

Research of EVs is currently focused on their function and molecular composition (miRNA, DNA or proteins), whereas studies of changes in size and/or concentration of EVs as biomarkers of disease are rare. This is likely due to EVs’ small size and low refractive index, making their detection and analysis technically very difficult^18,19^. Furthermore, many methods for quantification of small EVs do not recognize the nature of analyzed particles, thus any co-enriched non-EV particles can affect the accuracy of EV measurements^20^. Besides the direct determination of EVs' size by electron microscopy, several indirect methods exist where a large amount of collected data helps to determine EVs average size, size distribution and concentration in the sample^21^. Larger EVs are commonly measured using flow cytometry^20,22^, while for detection of smaller EVs^23–25^ nanoparticle tracking analysis (NTA), dynamic light scattering and resistive pulse sensing are more widespread. These methods were already used to quantify EVs in a limited number of biomarker studies^26–28^. Currently the most common tool for simple quantification of small EVs in the solution is NTA, where size and concentration of particles are calculated by capturing the amount of laser light scattering and the Brownian movement of particles in the solution on a single-particle level^20^. Recently, asymmetrical flow field-flow fractionation (AF4) started to gain relevance as a final size-fractionation step before analysis of size and concentration of particles in EV-enriched samples^29–31^. Historically, this technique was used for separation of proteins, polymers, viruses and lipoproteins based on size and speed of movement through the column^32^. When coupled to different detectors, like ultraviolet (UV) and multi-angle light scattering (MALS) detectors, the method enables quantification of EVs heterogeneous in size and can indirectly provide information on presence of contaminants high in protein or DNA content^31^. Altogether, EV-enrichment should be coupled to different quantification techniques, to provide sufficient dynamic range for reliable determination of EV size and concentration in biomarker discovery studies.

In this study we evaluated the suitability and repeatability of sedimentation rate- and size- based methods for enrichment of EVs from relatively small volumes of human blood plasma. Enriched EVs from ten healthy adult volunteers were analyzed for size, concentration and purity by NTA, AF4-UV-MALS, transmission electron microscopy (TEM), enzyme-linked immunosorbent assay (ELISA) and quantitative PCR (qPCR). To this end, AF4-UV-MALS was optimized for use on blood plasma EVs. Our aim was to find a simple method for enrichment of plasma EVs, compatible with reliable quantification of EVs as biomarkers of disease.

## Results

### sUC and SEC enrich different populations of nanoparticles from human plasma

In all study subjects, EVs were enriched from defrosted aliquots of cell- and larger EVs- depleted plasma using two methods, separating based on different particle characteristics: with in-lab prepared sUC and with commercially available SEC columns.

**Figure 1:**
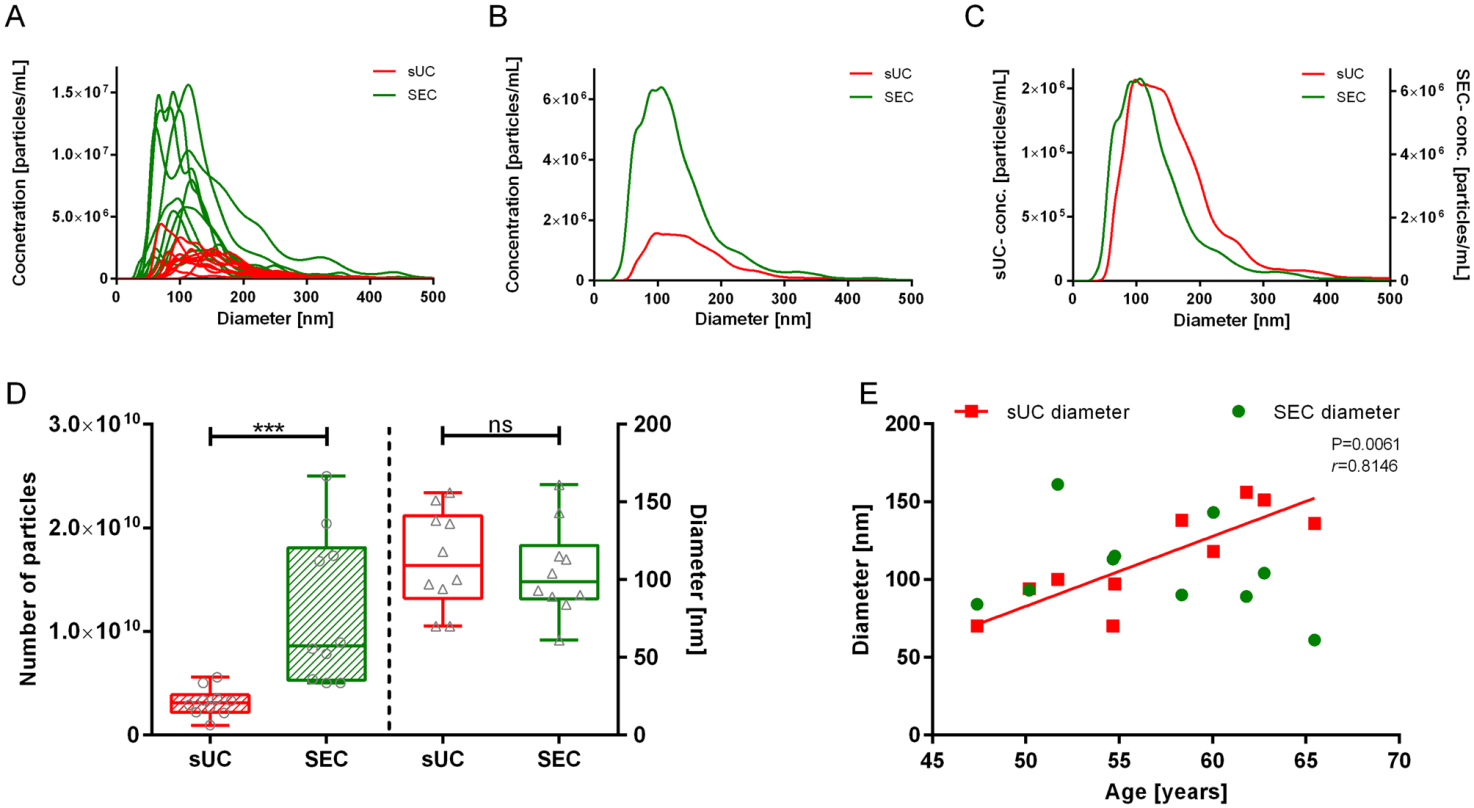
Quantification of enriched nanoparticles from human blood plasma by NTA. (**A**) NTA signal for samples of all included subjects after sUC and SEC. Comparison of the average particle (**B**) concentration and (**C**) size distribution for included subjects after sUC and SEC. (**D**) Box and whiskers plot of particle concentration and size in samples after sUC and SEC. Each circle or diamond presents one sample. ***p < 0.001. (**E**) Correlation of particle mode diameter after EVs-enrichment with age of the study subjects: SEC: *p* = 0.5603; sUC *p* = 0.0061, *r*_*s*_ = 0.8146. N = 10. *NTA* nanoparticle tracking analysis, *sUC* sucrose cushion ultracentrifugation, *SEC* size exclusion chromatography.

To quantify nanoparticles in the samples obtained from the above mentioned EV-enrichment methods, we first measured the mode size and concentration of nanoparticles by NTA for each study subject and calculated the median of those measurements (Fig. 1, Table 1). We observed significant (*p* < 0.001) differences in the NTA-concentration of nanoparticles between sUC and SEC EV-enrichment (Fig. 1A,B,D). Overall, sUC led to a statistically significant lower NTA-concentration of particles [3.11 (2.33–3.48) × 10^9^ particles/mL, median (25–75%), N = 10] than SEC [8.64 (6.00–17.16) × 10^9^ particles/mL, median (25–75%), N = 10]. However, variability in the NTA-concentration of nanoparticles between samples of included study subjects was larger after SEC (Fig. 1A,D). The median of size profiles of nanoparticles of all study subjects was comparable between two EVs-enrichment methods (Fig. 1C,D; sUC: 109 (95–138) nm, SEC: 99 (89–115) nm; median (25–75%), N = 10). Interestingly, only the diameter of nanoparticles in sUC samples statistically correlated with the age of the study subjects (Fig. 1E; *p* = 0.0061, *r*_*s*_ = 0.8146). Together these results imply a possible difference in the nature of nanoparticles in the samples after EV-enrichment with sUC and SEC methods.

**Table 1:** Concentration and size of nanoparticles, enriched by SEC or sUC, as measured by NTA and AF4-UV-MALS.

Sample	Sex	Age (years)	NTA				AF4-UV-MALS	
			SEC		sUC		sUC	
			Conc. of particles (particles/mL) (*10^9^)	Mode diameter (nm)	Conc. of particles (particles/mL) (*10^9^)	Mode diameter (nm)	Conc. of particles (particles/mL) (*10^9^)	2*R_geom_ (nm)
S1	F	51.7	5.39	161	5.04	100	0.19	190
S2	F	54.8	7.84	115	2.21	97	0.23	200
S3	F	62.8	8.93	104	3.36	151	0.23	212
S4	F	60.1	5.04	143	2.69	118	0.81	174
S5	M	47.4	16.80	84	0.97	70	NA	NA
S6	M	50.2	8.35	93	2.13	94	0.50	210
S7	M	61.8	17.28	89	3.31	156	NA	NA
S8	F	65.5	25.00	61	3.52	136	0.99	188
S9	F	58.4	5.04	90	2.91	138	0.92	212
S10	M	54.7	20.40	113	5.60	70	16.00	178
Median		56.6	8.64	99	3.11	109	0.66	195
1st–3rd quartile		52.5–61.4	6.00–17.16	89–115	2.33–3.48	95–138	0.23–0.94	186–211

*NTA* nanoparticle tracking analysis, *AF4-UV-MALS* asymmetrical flow field-flow fractionation with ultraviolet and multi-angle light scattering detectors, *SEC* size exclusion chromatography, *sUC* ultracentrifugation with sucrose cushion, *R*_*geom*_ geometric radius.

**Figure 2:**
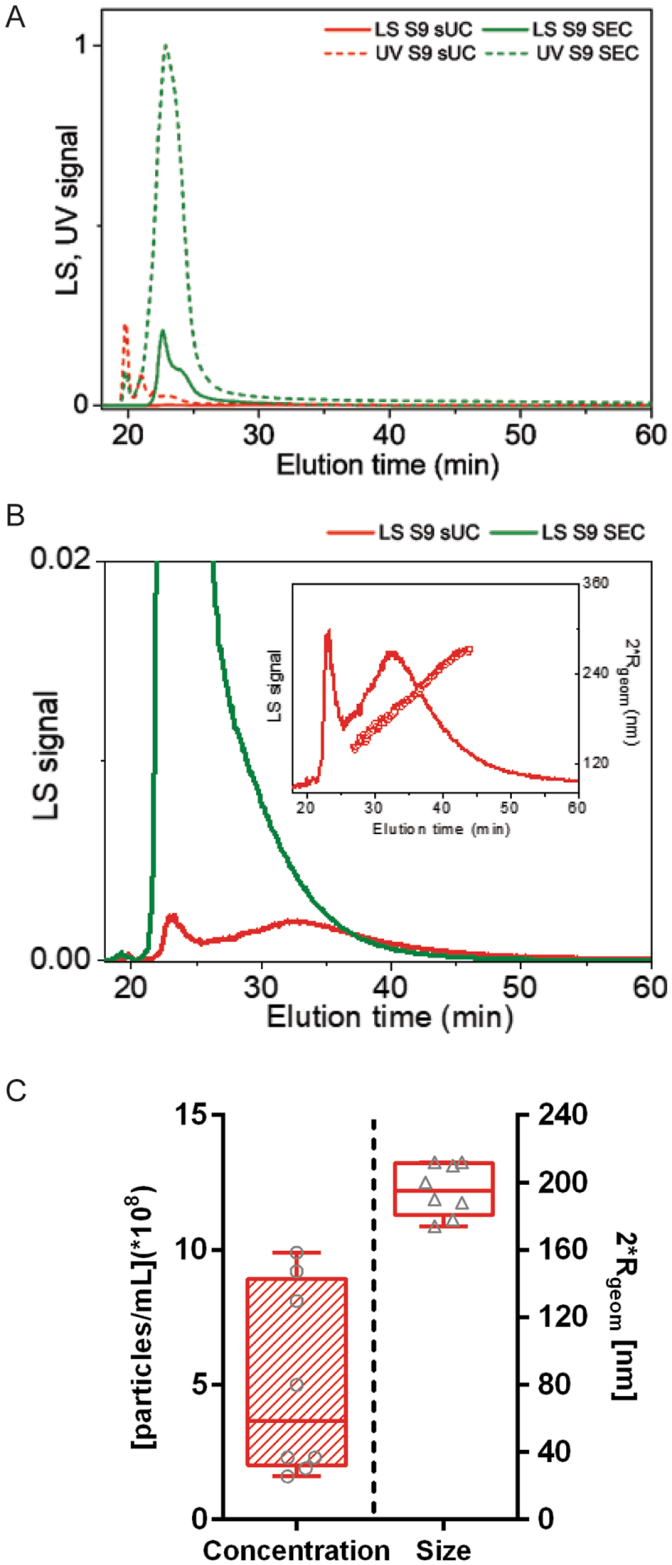
Quantification of enriched nanoparticles from human blood plasma by AF4-UV-MALS. (**A**) Representative fractogram. Representation of MALS (LS) and UV signals in relation to elution time in sample from subject 9 (S9) after sUC or SEC. Dotted lines present UV signals, solid lines present MALS signals. (**B**) Representative fractogram. Magnification of the MALS(LS) signal. The inset represents calculated size of eluted particles, presented as 2*R_*geom*_. (**C**) Particle size and concentration in samples after sUC. Each circle or triangle presents one sample. N = 8. *AF4-UV-MALS* asymmetrical flow field-flow fractionation with ultraviolet and multi-angle light scattering detectors, *sUC* sucrose cushion ultracentrifugation, *SEC* size exclusion chromatography, *R*_geom_ geometric radius.

All samples were further analyzed by AF4, coupled with UV and MALS detectors (Fig. 2). UV and MALS signal were very high and overlapped in EV-enriched samples after SEC, which made their quantification impossible (Fig. 2A). On the other hand, EV samples enriched by sUC could be reliably characterized, since UV and MALS signal were separated, and thus size and concentration of particles could be evaluated (Table 1, Fig. 2). Subpopulation of nanoparticles eluted between 25–58 min after analysis start (corresponding to 2*R_geom_ > 120 nm) were significantly larger compared to NTA measurements (2*R_geom_: 195 (186–211) nm, *p* < 0.0001; median (25–75%), N = 8), but also significantly less concentrated [0.66 (0.23–0.94) × 10^9^ particles/mL *p* < 0.01; median (25–75%), N = 8]. Results of nanoparticle quantification with NTA and AF4-UV-MALS, as well as sex and age of the study subjects, are presented in Table 1.

**Figure 3:**
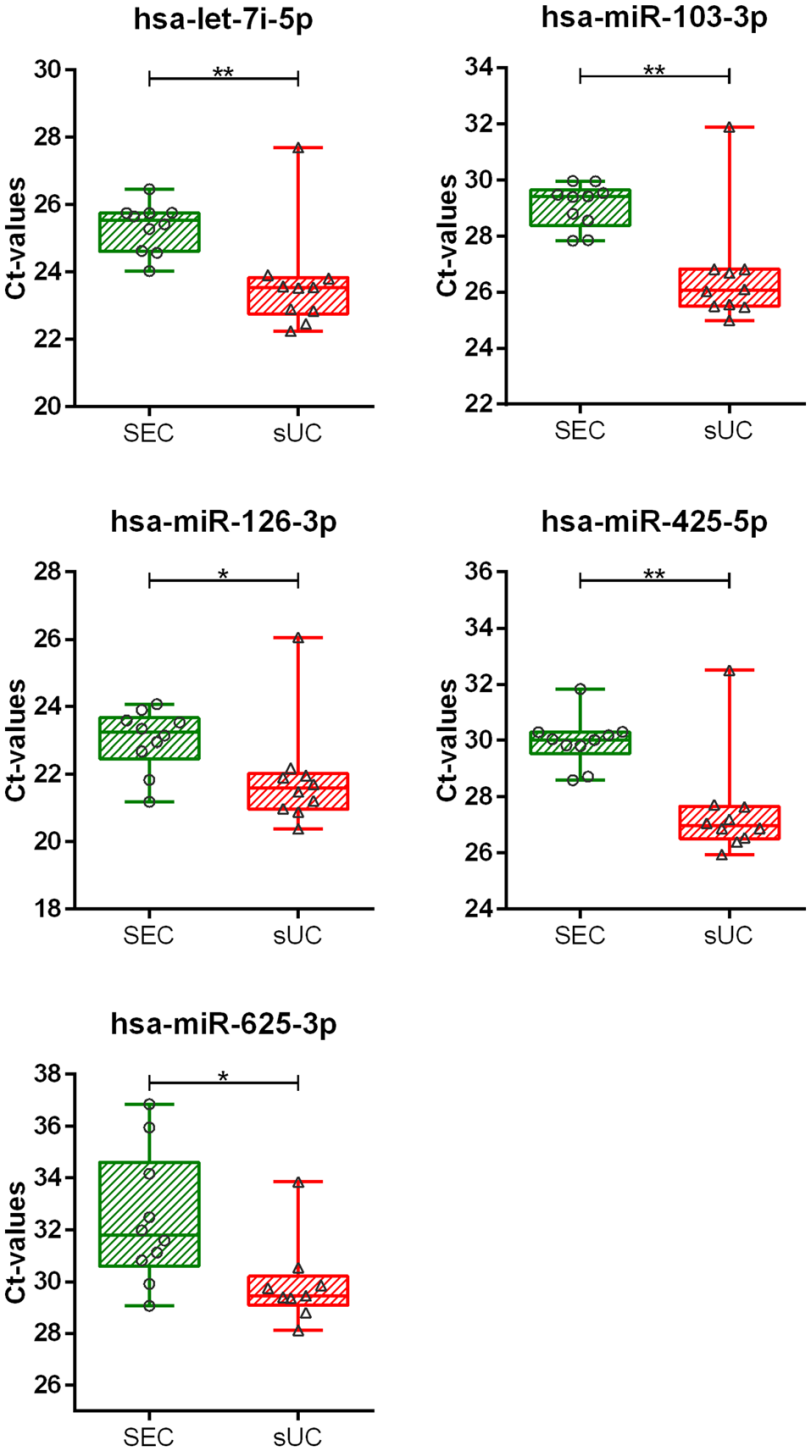
Quantification of enriched nanoparticles from human blood plasma by qPCR. Ct values of five selected miRNAs in samples after sUC and SEC. Each square or circle presents one sample. N = 10. *p < 0.05, **p < 0.01. *Ct* cycle threshold, *sUC* sucrose cushion ultracentrifugation, *SEC* size exclusion chromatography.

We additionally quantified nanoparticles in the EV-enriched samples indirectly by measuring expression (Ct) of miR-103a-3p, miR-126-3p, miR-425-5p, miR625-3p, and let-7i-5p, which were previously detected in EVs from human plasma^33^. SEC EV-enriched samples in general showed more variability in the Ct -values between samples of included study subjects than sUC EV-enriched samples (Fig. 3). Ct-values of all quantified miRNAs were significantly lower in samples after sUC compared to samples after SEC EV-enrichment (Fig. 3; *p* < 0.01 for all tested miRNAs, except for hsa-miR-126-3p and hsa-miR-625-3p: *p* < 0.05), indicating higher concentration of isolated miRNAs after sUC.

As shown by NTA, AF4–UV-MALS and qPCR sample characterization, sUC and SEC methods result in enrichment of different populations of nanoparticles. To determine the nature of said particles, we inspected EV-enriched samples under TEM.

### sUC EV-enrichment led to better removal of lipoprotein and protein contaminants in the sample

**Figure 4:**
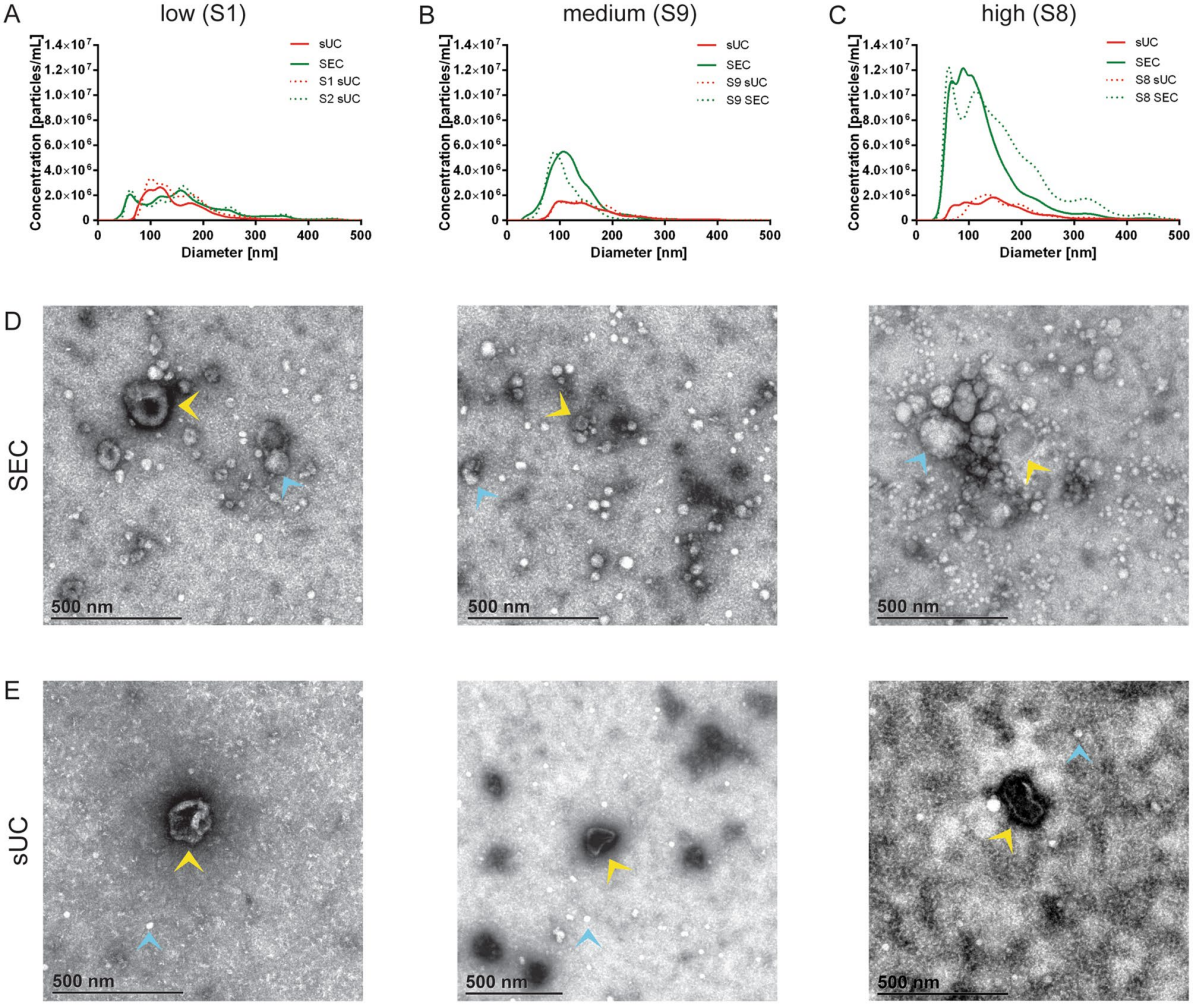
Comparison of representative TEM images of enriched nanoparticles from human blood plasma after SEC and sUC. (**A**) Comparison of NTA signal for average (solid line) and representative (dotted line) samples after sUC and SEC in samples with low, (**B**) medium and (**C**) high concertation of particles after SEC. (**D**) TEM images of representative samples after EVs enrichment with SEC. (**E**) TEM images of representative sample of each group after EVs enrichment with sUC. Yellow triangles indicate EVs, blue triangles indicate lipoproteins. *TEM* transmission electron microscopy, *NTA* nanoparticle tracking analysis, *sUC* sucrose cushion ultracentrifugation, *SEC* size exclusion chromatography.

TEM analysis of the EV-enriched samples showed that both enrichment methods isolated at least some EVs, but importantly differed in the amount of the co-isolated impurities (Fig. 4). For the purpose of this analysis, we separated subject’s samples into three groups based on NTA quantification of particles in SEC EV-enriched samples; those are groups with low (S1 and S4; Fig. 4A), medium (S2, S3, S6 and S9; Fig. 4B) and high (S5, S7, S8 and S10; Fig. 4C) particle concentration. Representative TEM images for all samples are collected in Supplementary Table 1. Beside nanoparticles that were determined as EVs, we observed high amounts of low electron lucent (white) amorphous non-EV particles of size up to 500 nm in the SEC (Fig. 4D), but not in the sUC samples (Fig. 4E). These nanoparticles were proposed to represent lipoproteins, based on their electron homogeneously lucent appearance due to very low binding of uranyl stain that is typical for lipids^34^. When present in higher concentrations as in SEC EV-enriched samples, lipoproteins tended to accumulate together and form larger structures, capturing protein aggregates and even EVs (Fig. 4D, Supplementary Table 1). TEM images of sUC EV-enriched samples showed relatively pure EVs, with few very small white spherical particles (10–30 nm, likely HDLs) and some protein aggregates still present (Fig. 4E, Supplementary Table 1).

**Figure 5:**
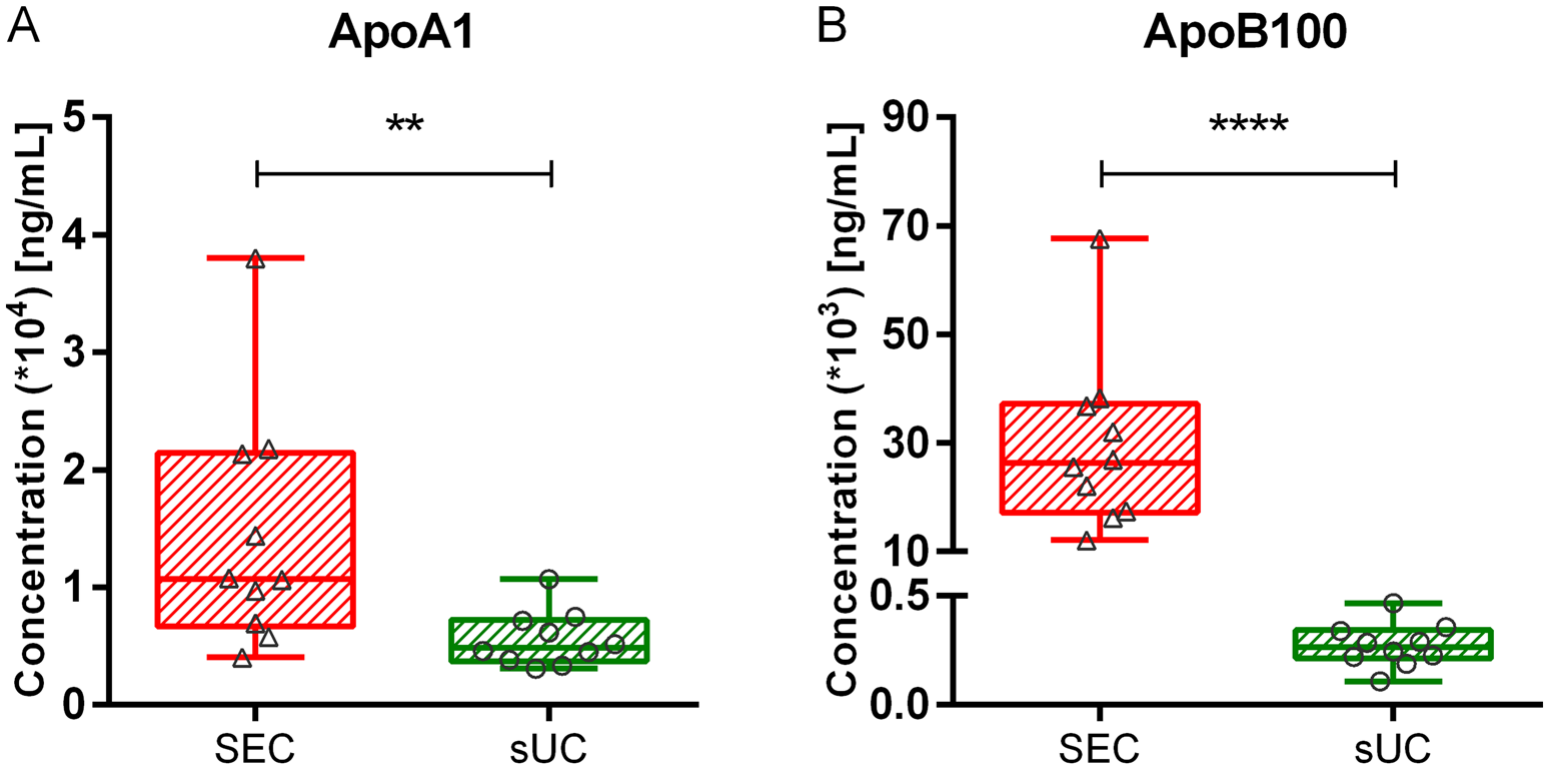
Quantification of ApoA1 and ApoB100 with ELISA in SEC and sUC EV-enriched samples. Box and whiskers plot of (**A**) ApoA1 and (**B**) ApoB100 concentrations in samples after SEC and sUC. Each circle or triangle presents one sample. N = 10. **p < 0.01, ****p < 0.0001. *ApoA1* Apolipoprotein A1, *ApoB100* Apolipoprotein B100, *sUC* sucrose cushion ultracentrifugation, *SEC* size exclusion chromatography.

To support TEM results, concentration of lipoproteins in SEC and sUC EV-enriched samples was measured by ELISAs specific for Apolipoprotein A1 (ApoA1, Fig. 5A) and Apolipoprotein B100 (ApoB100, Fig. 5B), characteristic for HDLs and LDLs, IDLs or VLDLs, respectively. Concentration of both lipoproteins was significantly higher in samples after SEC compared to sUC enrichment. While the difference in ApoA1 was approximately twofold [SEC: 1.070 (0.761–1.960) × 10^4^ ng/mL vs. sUC: 0.484 (0.399–0.689) × 10^4^ ng/mL, *p* < 0.01; median (25–75%), N = 10] the difference in ApoB100 was as much as 100-fold [SEC: 26.312 (18.618–35.610) × 10^3^ ng/mL vs. sUC: 0.263 (0.221–0.352) × 10^3^ ng/mL, *p* < 0.0001; median (25–75%), N = 10], signifying HDLs, but especially LDLs, IDL and VLDLs were present in much higher concentrations in samples after SEC. Results of ApoA1 and ApoB100 quantification are presented in Supplementary Table 2.

Despite higher concentration of nanoparticles in EV-enriched samples after SEC, high concentration of contaminat lipoproteins are co-purified. sUC method leads to high purity of EVs in the samples, and is therefore a more suitable method for enrichment of EVs from human plasma.

### sUC-based enrichment of EVs from plasma linked to NTA or AF4-UV-MALS quantification gives repeatable results

**Figure 6:**
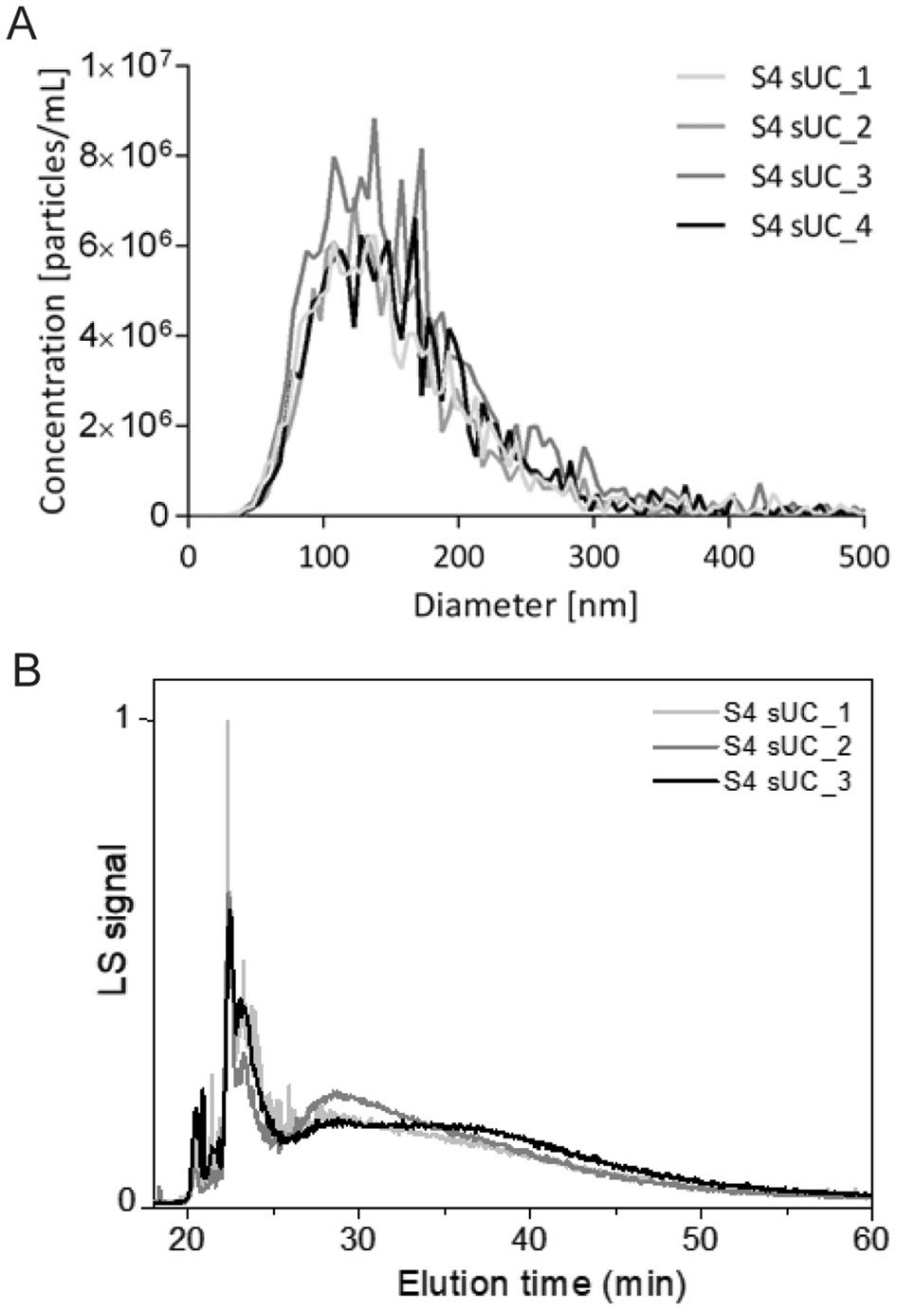
Repeatability of the sUC enrichment method linked to quantification with NTA and AF4-UV-MALS. (**A**) Quantification of nanoparticle size and concentration of four independently sUC-enriched aliquots of the same plasma sample by NTA. (**B**) Quantification of size and concertation of three independently sUC-enriched aliquots of the same plasma sample by AF4-UV-MALS. Representative fractogram. Representation of MALS and UV signals in relation to elution time in average of sample with and without enzymatic digestion after sUC. *NTA* nanoparticle tracking analysis, *AF4-UV-MALS* asymmetrical flow field-flow fractionation with ultraviolet and multi-angle light scattering detectors, *sUC* sucrose cushion ultracentrifugation.

We wanted to analyze the repeatability of sUC EV-enrichment method, linked to quantification of EVs by NTA (Fig. 6A, Supplementary Table 3) and AF4-UV-MALS (Fig. 6B, Supplementary Table 3). Percent relative standard deviation (%RSD), the measure of repeatability, was especially good when comparing the size of EVs, as the RSD was 5.4% when measured by NTA (mode diameter) and 2.1% when measured by AF4-UV-MALS (2*R_geom_). Percent RSD increased for EV concentration in the samples, as RSD was 19.1% and 24.4% when analyzed by NTA and AF4-UV-MALS, respectively.

**Figure 7:**
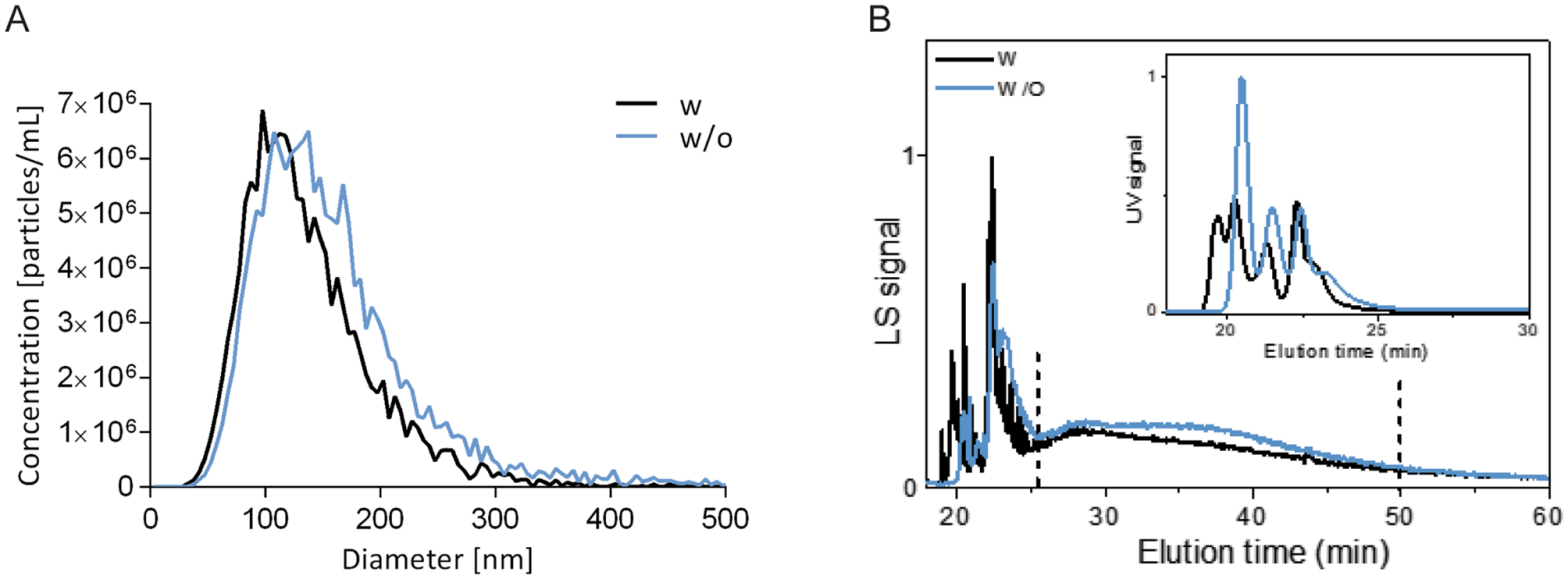
Effect of digestion with proteinase K and DNase-I on particle size and concentration after enrichment with sUC. (**A**) Quantification of size and concentration of nanoparticles human blood plasma sample with (black line) or without (blue line) treatment with proteinase K and DNase-I by NTA. Lines represent the average particle values of four independently sUC-enriched and treated aliquots of the same sample with and without enzymatic digestion. (**B**) Quantification of size and concentration of nanoparticles in human blood plasma sample with (black line) or without (blue line) treatment with proteinase K and DNase-I by AF4-UV-MALS. Fractograms present average MALS and UV signals in relation to elution time of three independently sUC-enriched samples with and without enzymatic digestion. *NTA* nanoparticle tracking analysis, *AF4-UV-MALS* asymmetrical flow field-flow fractionation with ultraviolet and multi-angle light scattering detectors, *sUC* sucrose cushion ultracentrifugation.

Next, we treated the EV-enriched samples with proteinase K and DNase-I to evaluate the influence of any remaining protein or DNA contaminants on EVs quantification by NTA (Fig. 7A, Supplementary Table 3) and AF4-UV-MALS (Fig. 7B, Supplementary Table 3). According to NTA, digestion did not considerably change the size profile, or significantly change the modal size (*p* = 0.0571) nor the concentration (*p* = 0.6857) of enriched EVs. Same general trend was seen for digested EV-samples after analysis with AF4-UV-MALS (modal size: *p* = 0.4286; concentration: *p* = 0.7143). Importantly, the efficiency of enzyme degradation was demonstrated by decrease and slight move to left of the UV-signal curve for digested samples on AF4-UV-MALS histogram (Fig. 7B).

The sUC EV-enrichment method linked to quantification with NTA or AF4-UV-MALS is repeatable, as determined by low %RSD in EVs size. Digestion of the EV-enriched samples with DNase-I and Proteinase K is redundant, as it does not significantly affect the quantification of EVs.

## Discussion

In search for new biomarkers, a simple method for enrichment of EVs from small starting volumes of human plasma is needed for their reliable quantification. We compared two methods for enrichment of EVs from one mL of blood plasma, separating particles based on their sedimentation rate—sUC, and size—SEC. According to NTA, AF4-UV-MALS, miRNA quantification, TEM and ELISA, sUC enrichment method leads to high purity of EVs in the samples. High nanoparticle concentrations after SEC resulted from substantial contamination with lipoproteins and other aggregates of EV-like sizes. We additionally showed that sUC EV-enrichment method, followed by quantification with NTA or AF4-UV-MALS, is repeatable and could in the future be used for studies of plasma EVs as biomarkers of disease.

Although commercially available SEC columns are promoted as compatible with EV-enrichment from the blood plasma^35,36^, in our hands they were not so effective for this purpose. SEC in our study co-enriched numerous non-EV contaminants as detected by NTA and AF4-UV-MALS. They were later identified as lipoproteins and lipo/protein aggregates by TEM and ELISAs specific for ApoA1 (found in HDL) and ApoB100 (found in LDL, IDL and VLDL). This is not surprising, as SEC method isolates nanoparticles based on size and many lipoproteins (LDLs, VLDLs and chylomicrons) and also larger protein aggregates^37^ are in the same size-range as EVs^38^. Moreover, lipoproteins are approximately 10^7^-fold more abundant in human blood plasma than EVs^7,9^, with reported concentrations of ApoA1 and ApoB100 in healthy adults of 189.11 × 10^4^ ng/mL and 147.25 × 10^4^ ng/mL, respectively^39^. We have shown that high concentrations of ApoA1 (median value: 1.1 × 10^4^ ng/mL) and ApoB100 (median value: 2.6 × 10^4^ ng/mL) are present in the sample even after enrichment of EVs with SEC. As lipoproteins can also stick to and aggregate with EVs^17^, they can considerably affect enrichment and quantification of EVs. Furthermore, as prandial status^40^, genetic makeup^41^ and lifestyle^42^ of study subjects affect plasma lipoprotein levels, any EV-enrichment method that cannot discard lipoproteins is hindered by these additional pre-analytical factors^43,44^. Despite high reported recovery of EVs with SEC^43^, the method is evidently less suitable for direct isolation of EVs from plasma. Some have addressed this by linking SEC to other EV-enrichment methods, like density gradient UC or highly specialized methods^45,46^, but these approaches can be complex, time-consuming, expensive and non-compatible with high numbers of low-volume samples, typical for clinical settings. Still, SEC is a valuable method for enrichment of EVs from other biological sources, where lipoproteins are not the main contaminant, like urine^47^ and cerebrospinal fluid^48^.

We therefore propose sUC, a simple method for one step enrichment of EVs from small volumes of blood plasma, which can be coupled to different methods for quantification of EVs. sUC allowed for simultaneous processing of many plasma samples towards final enrichment of EVs and important decrease in the number of larger lipoproteins (0.1% of normal plasma ApoB100 concentration) and aggregates, independent of prandial status of study subjects. Sucrose cushion UC was historically used for routine isolation of enveloped viruses^49,50^, which show similar biophysical characteristics as EVs^51^. It was also successfully used for enrichment of EVs from conditioned media^13,52,53^, but this is the first report of its use for enrichment of EVs from blood plasma. Although sUC enriched EVs still contained some HDLs as shown by TEM and confirmed by ELISA measurement of ApoA1 (median value: 0.48 × 10^4^ ng/mL), they do not interfere with EV quantification by NTA or AF4-UV-MALS, as they are only about 10–20 nm in diameter^54^. This is supported by our observation that EV diameter after sUC-enrichment correlates with study subjects’ age, but this should be confirmed in a larger study. HDLs were reported to transport miRNA^54,55^, but the results are not yet conclusive^56^. Still, when miRNA characterization is the end goal of the study, we propose separate quantification of HDLs in EV-enriched sample to evaluate their potential contribution to miRNA expression or treatment of EV-enriched sample with protease/RNase to release and degrade lipoprotein associated miRNAs, as proposed by Driedonks et al.^57^. Alternatively, HDLs can be removed from plasma by antibody-mediated clearing of lipoproteins^58^. Plasma EVs, enriched by UC-based methods, have previously been used in functional studies^10^, implying sUC does not impair EV’s functional activity.

Concentration of nanoparticles in the same EV-enriched sample was significantly lower when comparing AF4-UV-MALS to NTA quantification, while an average reported size of particle was almost twice as big in the case of AF4-UV-MALS. Although these methods detect and report different types of particle size^59^, a comparison between *R*_geom_ obtained by AF4-UV-MALS and *R*_h_ obtained by NTA is reasonable, since the vesicle’s scattering mass is distributed on a thin shell surrounding the spherical core^60^. However, the methods seem to detect a different subpopulation of nanoparticles in the EV-enriched sample. Even though NTA is in theory suited for polydispersed samples, only EVs larger than approximately 60 nm in diameter are detected^61^, furthermore EVs cannot be distinguished from lipoproteins or larger protein aggregates with similar Brownian motion^10,62^. Additionally, a previous study showed NTA prevalently reports properties of approximately 200 nm-sized particles, regardless of sample actual composition^63^. On the other hand, AF4-UV-MALS is known to detect a broad size range of particles (1 nm–10 µm)^29^, also due to initial separation of particles before they reach detectors. Still, most of smaller particles were not included in particle radius calculations in our study, as they co-eluted with other contaminants that absorb UV light. Our^31^ and other studies namely showed that UV detector at 280 nm measures proteins^64^ and lipoproteins^65^ in the sample. To exclude the effect of impurities on EV quantification, we effectively included in the AF4-UV-MALS analysis only particles with 2*R_geom_ larger than ≥ 120 nm. NTA and AF4-UV-MALS are evidently great complementary techniques for quantification of EVs from a complex source.

Importantly, we demonstrated good repeatability of the sUC-based enrichment of EVs from human plasma, when linked to quantification by NTA and AF4-UV-MALS. Percent RSD of EV size, measured in independently processed samples from the same plasma source, was namely 5.4% and 2.1% when analyzed by NTA or AF4-UV-MALS, respectively. We observed higher %RSD of EV concentration in processed samples (19.1% and 24.4% for NTA and AF4-UV-MALS, respectively), but this is likely due to inherent limitations of the instruments used for quantification. Higher %RSD is namely reported for measurements of EV concentration by NanoSight NS300 when compared to %RSD for EV size (5.4–10.7% for concentration vs. 0.8–6.7% for size)^61^. A similar study for AF4-UV-MALS has not yet been performed. Development and introduction of validated EVs reference materials, such as recombinant EVs, will in the future allow further normalization of each sample for its specific recovery efficiency^45^, leading to even better repeatability and methodological standardization of the protocol.

This is to our knowledge one of the first reports of analysis of human blood plasma EVs by AF4-UV-MALS. One study directly analyzed proteins, antigens and smaller lipoproteins in diluted rat plasma and human whole blood by AF4-UV-MALS^64^, while a recent study successfully analyzed EVs in pre-processed plasma of human subjects that were fasted for at least 12 h^66^. We have previously implemented AF4-UV-MALS for quantification of EVs by using commercially available EV standards^31^ and monodisperse polystyrene beads of various radii^67^. The technique was later adopted by others in many quantification studies of cell culture- and urine-derived EVs^13,29,30,36,47^. Different biological sources of EVs each have distinct nanoparticle composition, with different contaminants possibly interfering with EV quantification. As shown here, the biggest challenge of blood-derived EVs were lipoproteins^30,47^. MALS signal for particles and UV signal for present lipoprotein impurities completely overlapped in the SEC EV-enriched samples, so quantification was only possible for the subpopulation of sUC enriched EVs, where UV signal was low. Wu et al.^66^ addressed this problem by using at least 12 h fasted plasma, but this is not always possible in the clinical setting and in the context of certain diseases. Efficiency of AF4-UV-MALS therefore still seems to greatly benefit from carefully chosen method for enrichment of EVs, like here described sUC.

In conclusion, we propose sUC EV-enrichment method, coupled to NTA or AF4-UV-MALS, for simple and repeatable quantification of human plasma EVs in biomarkers studies. Optimization of AF4-UV-MALS for quantification of EVs from blood plasma opens new possible clinical utility for the technique. In the future, sUC EV-enrichment method should be tested on larger cohorts in connection to different diseases.

## Methods

### Study subjects

Ten healthy adult volunteers (median age of 56.5 years at enrolment) were included in the study, which was approved by the Slovenian Ethics Committee for Research in Medicine and was carried out according to the Declaration of Helsinki. Each participant signed an informed consent.

Peripheral whole venous blood of unfasted volunteers was collected into commercially available vacutainer tubes containing EDTA (BD Vacutainer, BD, USA) in the morning and processed within 1 h after withdrawal. After the first centrifugation (820×*g* for 10 min at 4 °C) plasma was collected and centrifuged again (2500×*g* for 10 min at 4 °C). The supernatants were then aliquoted to protein low binding tubes (Eppendorf, Germany) and stored at − 80 °C. Before small EV enrichment, aliquots were defrosted on ice and centrifuged at 10,000×*g* for 20 min at 4 °C to remove large nanoparticles.

### Small EV-enrichment with sucrose cushion ultracentrifugation (sUC)

One mL of plasma was diluted with 8.5 mL particle-free Dulbecco’s Phosphate buffer saline (dPBS, Sigma-Aldrich, USA), carefully overlaid on 2 mL of 20% sucrose cushion (sucrose (Merck Millipore, USA) in dPBS) in polypropylene tubes (Beckman Coulter, USA) and centrifuged at 100,000×*g* for 135 min at 4 °C (MLA-55, Beckman Coulter, USA) to enrich EVs in the sample. The supernatant was gently aspirated and residual moisture absorbed from the walls of the tubes. The pellet was resuspended in small volume of dPBS (NTA: 20 µL, AF4-UV-MALS: 40 µL, TEM: 20 µL) and stored at − 20 °C. For miRNA extraction, pellet was resuspended in 200 µL of dPBS and 800 µL Tri-reagent (Sigma, USA) was added before storage.

To analyze repeatability of sUC EV-enrichment method linked to quantification methods, 4 aliquots of plasma from the same donor were processed as described above. Repeatability is measured by % relative standard deviation (%RSD) and is calculated as: (standard deviation/mean)*100.

### Small EV-enrichment with size exclusion chromatography (SEC)

Five hundred µL of plasma was loaded on the SEC column (qEV original, IZON Science, USA) and eluted with dPBS. Twenty eluted fractions of 500 µL were collected in protein low binding tubes. After flushing the column with 10 mL of fresh dPBS, the process was repeated with the remaining 500 µL of plasma as before. Based on A_280_ chromatogram (Synergy 2, BioTek, USA) proteins started to elute in fraction 12, with the bulk released in fractions 15–19. Therefore, only fractions 7–11, with enriched EVs, were collected (final volume = 5 mL) and concentrated (NMWL membrane, 100 kDa, Amicon Ultra-4 Centrifugal Filter Unit, Merck Millipore, USA) to 40 µL. Samples were further diluted depending on subsequent analysis as described for sUC.

### Quantification of EVs by NTA

EV-enriched samples were diluted in dPBS to a concentration in the range of 1 × 10^7^–10^9^/mL and examined using a NanoSight NS300 (NanoSight Ltd., UK) equipped with a 488 nm blue laser. Five 60 s-long videos were taken for each sample, with the best three chosen for analysis after visual inspection. Raw data of laser scattering and particle movement were analyzed using NTA software (version 3.3; NanoSight Ltd., UK). Automatic settings were selected for minimum expected particle size and blur, minimum track length was set to 10, detection threshold to 5, sample viscosity to the corresponding viscosity for water and temperature to 25 °C. The output data was presented as EV size (modal hydrodynamic diameter in nm) and EV concentration (number of EVs enriched from 1 mL of plasma in particles/mL).

### Quantification of EVs by AF4-UV-MALS

The AF4 was performed using the Eclipse 3+ system (Wyatt Technology Europe, Germany) connected to the isocratic pump, on-line vacuum degasser and auto-sampler (Agilent Technologies 1260 series, USA). The samples were separated in a trapezoidal-shaped channel, with a tip-to-tip length of 152 nm and an initial channel breadth of 21 mm that decreased to final 3 mm, equipped with the 350 µm spacer and 10 kDa regenerated cellulose membrane. The fractionated particles were detected with an on-line UV detector operating at 280 nm (Agilent Technologies, USA) and a multi-angle light scattering (MALS) detector (DAWN HELEOS, Wyatt Technology, USA) operating at a wavelength of 658 nm, calibrated using toluene and normalized with bovine serum albumin protein as an isotropic scatterer standard. As a running eluent, dPBS supplemented with 0.02% w/v sodium azide was used. The eluent was filtered through a Nylon 66 membrane with a pore-size of 0.45 µm (Supelco Analytical, USA). Between the HPLC pump and the AF4 channel, an additional filter with a pore-size of 0.1 µm was placed (PEEK Inline Filter Holder, Wyatt Technology, USA). Samples were injected during a focusing/injection step of 5 min, with a focus flow rate of 1.5 mL/min and an injection flow rate of 0.2 mL/min, followed by a focusing/relaxation step for an additional 7 min. The elution step was performed with two linear cross-flow gradients, a high initial gradient of 0.275 mL/min^2^ (i.e., cross-flow from 3.0 to 0.25 mL/min in 10 min) followed by a lower gradient of 0.0036 mL/min^2^ (cross-flow from 0.25 to 0.09 mL/min in 45 min). Here, 0.09 mL/min was the lowest cross-flow limit of the instrument. Once this limit was reached, the cross-flow was turned off. The detector flow rate was 1 mL/min. The last step was washing the channel for 10 min in elution mode without any cross-flow^13,31^.

The Astra 5.3.4.20 software (Wyatt Technology, USA) was used to analyze the data. The size of the particles was expressed as geometric radius (*R*_geom_). The *R*_geom_ was obtained from the MALS detector without the need for the solute concentration and the increment in the sample refractive index and was determined by the Astra Particle template assuming spherical EV shape^68^. The concentration of particles (particles/mL) was evaluated by the Astra Number density template, using an EV refractive index of 1.39^69^.

### DNase-I and proteinase K digestion

After sUC, the EV-enriched pellet was resuspended in 40 µL of DNase-I buffer (Thermo Scientific, USA) and transferred to a fresh protein low binding tube. First, 4.5 U of DNase-I (Thermo Scientific, USA) was added, gently mixed and incubated at 37 °C for 30 min. The activity of DNase-I was stopped by addition of ethylenediaminetetraacetic acid (final concentration 1 mM; Thermo Scientific, USA,). Next, samples were digested with Proteinase K (final concentration 50 μg/mL; Ambion, USA) for 30 min at 37 °C. After the digestion, Proteinase K was inhibited by incubation with 1 µL of Protease Inhibitor Cocktail (I3911, Sigma-Aldrich, USA) for 10 min at RT. Digested EV-enriched samples were stored at − 20 °C until use.

### EV-miRNA quantification

For isolation of miRNA, 1 mL aliquots of EV-enriched samples mixed with Tri-reagent, were defrosted on ice and mixed with 1 µL of SPIKE-IN (ath-miR-159a, final concentration 2 nM; Applied Biosystems, USA), 1 µL of MS2 RNA carrier (final concentration 0.8 µg/µL; Roche, Switzerland), 200 µL of chloroform (Sigma-Aldrich, USA) and mixed vigorously. miRNA was extracted by chloroform extraction, using the miRNeasy Mini Kit (Qiagen, Germany), according to the manufacturer’s instructions. The protocol was adapted by addition of extra chloroform extraction step (addition of 500 µL of RNase/DNase-free water after removal of aqueous phase from the chloroform-sample mixture) and changes in miRNA column elution step (two sequential additions of 25 µL of RNase/DNase free water and centrifugations at 15.000×*g*, 30 s). Eluted miRNA was stored at − 20 °C in DNA low binding tubes (Eppendorf, Germany).

Reverse transcription of miRNA to cDNA was performed in a batch (for all samples at once), transcribing total isolated miRNA using the TaqMan Advanced miRNA cDNA Synthesis Kit (Applied Biosystems, USA) and following manufacturers recommendations*.* Samples were stored at − 20 °C.

qPCR for miRNA expression analysis was performed using the TaqMan Advanced MicroRNA assay (Applied Biosystems, USA) on Viia 7 Real-Time PCR System (Applied Biosystems, USA). In addition to five miRNAs of interest (hsa-miR-103a-3p, hsa-miR-126-3p, hsa-miR-425-5p, hsa-miR625-3p, and hsa-let-7i-5p), threshold cycle (Ct) of spike-in was analyzed for efficiency of miRNA isolation and transcription to cDNA and deviations would result in exclusion of the sample. All samples were also tested for hemolysis by analyzing miR-23a-3p and miR-451a, ∆Ct (Ct_(miR-23a-3p)_ – Ct_(miR-451a)_) ≥ 7 indicated hemolysis and would result in exclusion of the sample. The analysis was performed using Viia 7 Software (Applied Biosystems, USA) and the miRNA levels expressed as Ct.

### Characterization of EVs by TEM

Samples were visualized by TEM using a negative staining method. Four µL of EV-enriched samples were applied on Formvar-coated and carbon stabilized copper grid and stained with 1% (w/v) water solution of uranyl acetate. Samples were analyzed with Philips CM 100 electron microscope (FEI, The Netherlands), operating at 80 kV. Images were obtained with OriusSC200 camera (Gatan, USA) using DigitalMicrograph Software (Gatan Inc., USA)^13^.

### Quantification of ApoA1 and ApoB100 with ELISA

EV-enriched samples were diluted in dPBS (100,000-times for measurements of ApoA1 in all samples, 3000-times for measurements of ApoB100 in samples after SEC and 80-times for measurements of ApoB100 in samples after sUC) and the concentration (ng/mL) of ApoA1 (specific for HDLs) and ApoB100 (specific for IDLs, LDLs and VLDLs) measured in duplicates by designated ELISAs (ELISA-ApoA1: #3710-1HP-2, ELISA-ApoB100: #3715-1HP-2, Mabtech, Sweden), according to the manufacturer’s instructions. Measured concentrations were normalized to 1 mL of starting plasma and any potential dilution of the samples was accounted for.

### Statistics

Data analysis and graphical presentations were performed using GraphPad Prism version 6.07 (GraphPad Software, USA). The Mann–Whitney test was used to test for differences between the two groups. Correlations between experimental results were examined using Spearman's rank test. *p* values of less than 0.05 indicated statistical significance.

## Supplementary information

Supplementary Tables.
