# Supplementary material for: Enrichment of plasma extracellular vesicles for reliable quantification of their size and concentration for biomarker discovery
Source: Sci Rep. 2020 Dec 7;10:21346. doi: 10.1038/s41598-020-78422-y (PMC7721811; doi:10.1038/s41598-020-78422-y)
Supplement: Supplementary file 1 — Supplementary Tables. [file 41598_2020_78422_MOESM1_ESM.pdf]

Enrichment of plasma extracellular vesicles for reliable quantification of their size and concentration for biomarker discovery

Marija Holcar[1], Jana Ferdin[1], Simona Sitar[2], Magda Tušek-Žnidarič[3], Vita Dolžan[1], Ana Plemenitaš[1], Ema Žagar[2], and Metka Lenassi[1]*

[1] Institute of Biochemistry, Faculty of Medicine, University of Ljubljana, Ljubljana, Slovenia, EU

[2] Department of Polymer Chemistry and Technology, National Institute of Chemistry, Ljubljana, Slovenia, EU

[3] Department of Biotechnology and System Biology, National Institute of Biology, Ljubljana, Slovenia, EU

Supplementary Table 1: Transmission electron microscopy micrographs of each sample, isolated either by sUC or SEC enrichment methods

Sample	SEC	sUC	Sample	SEC	sUC
**S1**	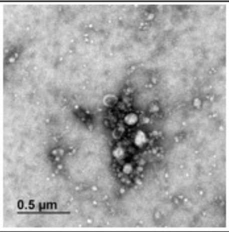	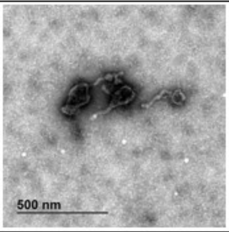	**S6**	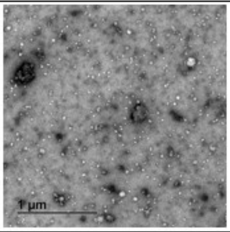	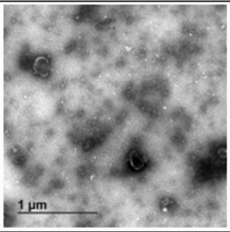
**S2**	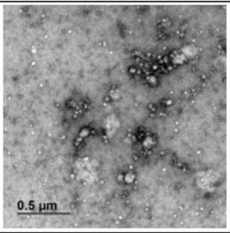	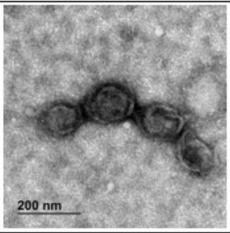	**S7**	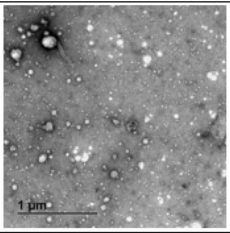	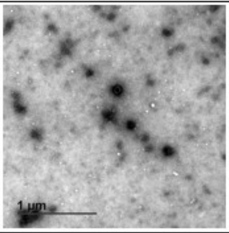
**S3**	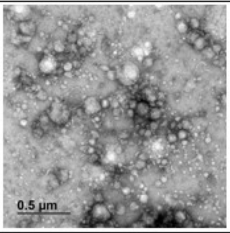	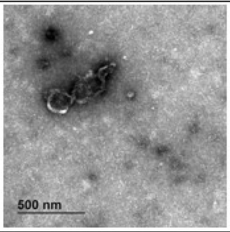	**S8**	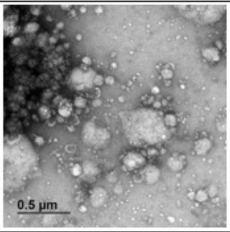	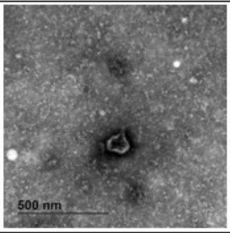
**S4**	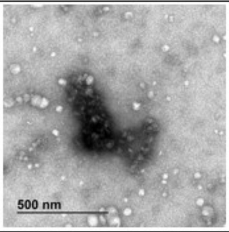	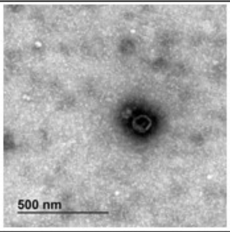	**S9**	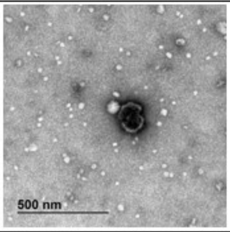	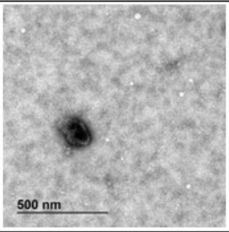
**S5**	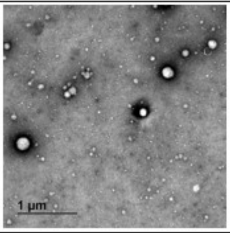	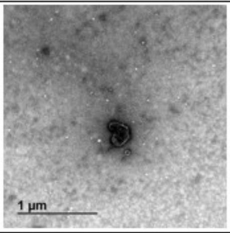	**S10**	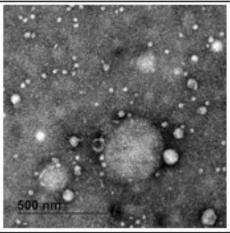	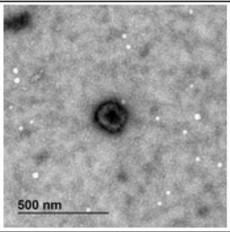

SEC – size exclusion chromatography, sUC- sucrose cushion ultracentrifugation

Supplementary table 2: Results of ApoA1 and ApoB100 analysis by ELISA in samples after sUC and SEC

	SEC		sUC	
**Sample**	Conc. of ApoA1 [ng/mL] (*10^4)	Conc. of ApoB100 [ng/mL] (*10^3)	Conc. of ApoA1 [ng/mL] (*10^4)	Conc. of ApoB100 [ng/mL] (*10^3)
**S1**	3.800	67.669	0.714	0.225
**S2**	1.077	12.151	0.306	0.188
**S3**	0.972	16.275	0.449	0.220
**S4**	1.064	26.976	0.330	0.107
**S5**	0.574	25.648	0.455	0.283
**S6**	2.178	31.968	0.747	0.356
**S7**	0.691	38.276	0.383	0.289
**S8**	1.437	36.824	0.513	0.242
**S9**	2.135	22.086	0.615	0.337
**S10**	0.400	17.463	1.068	0.466
**Median**	1.070	26.312	0.484 **	0.263 ****
**1st-3rd quartile**	0.761 - 1.960	18.618 – 35.610	0.399 - 0.689	0.221 – 0.325

**p<0.01, ****p< 0.0001, ApoA1 - Apolipoprotein A1, ApoB100 – Apolipoprotein B100, sUC - sucrose cushion ultracentrifugation, SEC - size exclusion chromatography.

Supplementary table 3: Concentration and size of extracellular particles, enriched by sUC with or without enzymatic digestion, as measured by NTA and AF4UV-MALS

	NTA				AF4-UV-MALS			
	w/o digestion		w digestion		w/o digestion		w digestion	
**Sample**	Conc. of particles [particles /mL] (*10^9)	Mode diameter [nm]	Conc. of particles [particles /mL] (*10^9)	Mode diameter [nm]	Conc. of particles [particles /mL] (x10^9)	2*R_{geom} [nm]	Conc. of particles [particles /mL] (x10^9)	2*R_{geom} [nm]
**S4 sUC_1**	2.16	114.3	2.90	126.6	/	/	0.44	196.0
**S4 sUC_2**	2.01	125.7	0.83	127.8	0.40	200.0	0.50	198.0
**S4 sUC_3**	2.91	111.8	2.36	115.3	0.60	198.0	0.42	196.0
**S4 sUC_4**	2.00	114.1	1.35	129.0	0.46	206.0	0.40	210.0
**Mean**	2.27	116.5	1.86	124.7	0.42	201.3	0.44	200.0
**%RSD**	19.07	5.4	50.55	5.1	24.44	2.1	9.82	3.4
**p value***			0.6857	0.0571			0.7143	0.4286

NTA - Nanoparticle tracking analysis, AF4 - asymmetrical flow field-flow fractionation with ultraviolet and multi-angle light scattering detectors, R_{geom} – geometric radius, RSD – relative standard deviation,*comparing results w/o digestion to results w digestion.
